# Anticancer efficacy of the hypoxia‐activated prodrug evofosfamide is enhanced in combination with proapoptotic receptor agonists against osteosarcoma

**DOI:** 10.1002/cam4.1115

**Published:** 2017-08-10

**Authors:** Vasilios Liapis, Aneta Zysk, Mark DeNichilo, Irene Zinonos, Shelley Hay, Vasilios Panagopoulos, Alexandra Shoubridge, Christopher Difelice, Vladimir Ponomarev, Wendy Ingman, Gerald J. Atkins, David M. Findlay, Andrew C. W. Zannettino, Andreas Evdokiou

**Affiliations:** ^1^ Discipline of Surgery Breast Cancer Research Unit Basil Hetzel Institute and Centre for Personalised Cancer Medicine University of Adelaide Woodville South Australia Australia; ^2^ Vascular Biology and Cell Trafficking Laboratory Centre for Cancer Biology University of South Australia Adelaide South Australia Australia; ^3^ Department of Radiology Memorial Sloan‐Kettering Cancer Center New York New York; ^4^ Discipline of Surgery School of Medicine at The Queen Elizabeth Hospital University of Adelaide Woodville Australia; ^5^ Robinson Research Institute University of Adelaide Adelaide Australia; ^6^ Discipline of Orthopaedics and Trauma University of Adelaide Adelaide South Australia Australia; ^7^ School of Medical Sciences Myeloma Research Laboratory Cancer Theme South Australian Health and Medical Research Institute (SAHMRI) Faculty of Health Science University of Adelaide Adelaide Australia

**Keywords:** Drozitumab, dulanermin, evofosfamide, hypoxia, osteosarcoma

## Abstract

Tumor hypoxia is a major cause of treatment failure for a variety of malignancies. However, hypoxia also leads to treatment opportunities as demonstrated by the development of compounds that target regions of hypoxia within tumors. Evofosfamide is a hypoxia‐activated prodrug that is created by linking the hypoxia‐seeking 2‐nitroimidazole moiety to the cytotoxic bromo‐isophosphoramide mustard (Br‐IPM). When evofosfamide is delivered to hypoxic regions of tumors, the DNA cross‐linking toxin, Br‐IPM, is released leading to cell death. This study assessed the anticancer efficacy of evofosfamide in combination with the Proapoptotic Receptor Agonists (PARAs) dulanermin and drozitumab against human osteosarcoma in vitro and in an intratibial murine model of osteosarcoma. Under hypoxic conditions in vitro, evofosfamide cooperated with dulanermin and drozitumab, resulting in the potentiation of cytotoxicity to osteosarcoma cells. In contrast, under the same conditions, primary human osteoblasts were resistant to treatment. Animals transplanted with osteosarcoma cells directly into their tibiae developed mixed osteosclerotic/osteolytic bone lesions and consequently developed lung metastases 3 weeks post cancer cell transplantation. Tumor burden in the bone was reduced by evofosfamide treatment alone and in combination with drozitumab and prevented osteosarcoma‐induced bone destruction while also reducing the growth of pulmonary metastases. These results suggest that evofosfamide may be an attractive therapeutic agent, with strong anticancer activity alone or in combination with either drozitumab or dulanermin against osteosarcoma.

## Introduction

Osteosarcoma (OS) is the most common primary type of cancer that develops in the bone and accounts for 20% of all primary osseous neoplasms 1, 2. Most OS occur in young adults and children and usually develop in areas where the bone is rapidly growing such as the proximal tibia, distal femur, and proximal humerus 3. Metastatic spread of OSs preferentially occurs in the lungs which is correlated with poor survival and is seen in 20% of patients with OS 4, 5. Over the past 20 years, treatment of OS has advanced considerably due to the increased efficacy of conventional chemotherapeutic agents. The type, combination as well as the doses of chemotherapeutic agents given as well as the sensitivity of the tumor cells determine the patients’ response to treatment. Despite these advances in treatment, drug resistance still remains a problem 6. In addition, conventional chemotherapeutic drugs have a significant impact on normal bone health, leading to a greater risk of developing osteoporosis and myelosuppression due to toxicities in the bone marrow 7, 8.

The characteristics of bone lesions caused by OS are based on their radiologic appearance which can be either osteoblastic (osteosclerotic), osteolytic, or a combination of both 9. Osteolysis is common with OS and is caused by the bone resorbing activity of osteoclasts 10, 11. Tumor growth is stimulated by factors released from the bone and in turn tumor cells produce factors that stimulate osteoclastic bone resorption, resulting in a mutual relationship of bone destruction between the cell types known as “the vicious cycle” 12. In contrast, osteoblastic lesions are associated with tumor cells that stimulate osteogenesis 13, 14.

As with most solid tumors, early‐stage OS displays significant regions of hypoxia, where resistant tumor cells reside, which results in tumor recurrence and metastasis, leading to treatment failure and poor outcomes. These hypoxic conditions found in tumor subregions are rarely observed in normal tissue; tumor hypoxia can therefore provide the basis for selective cancer therapy and there are a number of strategies currently being investigated to selectively target tumor cells in this hypoxic environment. Hypoxia‐activated prodrugs (HAPs) selectively deliver cytostatic or cytotoxic agents to hypoxic subregions.

Evofosfamide (formerly TH‐302) is a hypoxia‐activated prodrug composed of 2‐nitroimidazole linked to bromo‐isophosphoramide mustard (Br‐IPM) 15. The 2‐nitroimidazole component of evofosfamide serves as an oxygen sensor, releasing the crosslinking DNA‐alkylating Br‐IPM into the hypoxic regions of tumors. To date, evofosfamide has been investigated both as a stand‐alone agent and in combination with chemotherapy and other targeted cancer drugs against numerous solid tumor types and blood cancers 16, 17, 18.

Proapoptotic Receptor Agonists (PARAs), either as monotherapy or in combination with other agents, are generally well‐tolerated by patients with very few side effects 19 and although phase 1/1b studies provided encouraging preliminary results, findings from randomized Phase 2 studies failed to demonstrate significant clinical benefit 20, 21, 22, 23. Despite these clinical observations, there has been no investigation examining the anticancer efficacy of evofosfamide alone or in combination with either the Proapoptotic Receptor Agonists (PARAs) dulanermin (formerly known as Apo2L/TRAIL), or drozitumab for the treatment of osteosarcoma.

This study investigates the cytotoxic activity of evofosfamide alone and in combination with dulanermin and drozitumab against human OS cells in vitro and in vivo, using a clinically relevant orthotopic mouse model of OS and on their subsequent lung metastases.

## Materials and Methods

### Cells

The human OS cell lines BTK‐143 and K‐HOS were obtained from ATCC (Manassas, VA) and were authenticated by DNA (STR) profiling. Cells were cultured in Dulbecco's Modified Eagle's Medium (DMEM), supplemented with 2 mmol/L glutamine, 100 IU/mL penicillin, 160 *μ*g/mL gentamicin, and 10% fetal bovine serum (Life Technologies, Carlsbad, CA) in a 5% CO_2_‐containing humidified atmosphere. The generation of luciferase‐tagged BTK‐143‐TGL has been described previously 24.

Normal human osteoblasts (NHB) were obtained from bone marrow aspirations from the iliac crest of normal healthy donors or from the trabecular bone of osteoarthritic patients at joint replacement surgery, grown in *α*MEM (SIGMA, Saint Louis, Missouri) containing L‐ascorbic acid 2‐phosphate (Life Technologies, Carlsbad, CA) and 10% fetal bovine serum. Medium was then replaced at 4‐day intervals, cells were then consequently subcultured by treatment with a (0.1%) (w/v) mixture of collagenase and dispase. In all experiments, cells from the first passage were used in all experiments.

### Drugs

Threshold Pharmaceuticals (South San Francisco, CA) provided the evofosfamide powder which was dissolved in a sterile saline solution at a concentration of 13.2 mmol/L. The Caspase Inhibitor‐1 ZVAD‐fmk, was purchased from Calbiochem Inc. (La Jolla, CA). Both drozitumab and dulanermin were a gift from Dr Avi Ashkenazi, Genentech, Inc. (South San Francisco, CA). Affinity Pure Goat anti‐human IgG Fc*γ* fragment was purchased from Jackson Immuno Research Laboratories Inc. (West Grove, PA).

### Cell viability assay

To determine the cytotoxicity of evofosfamide on cell growth, 1 × 10^4^ cells per well were seeded in 96‐well microtiter plates and allowed to attach overnight. Cells were then treated with increasing concentrations of evofosfamide (1–100 *μ*mol/L) alone and in combination with 100 ng/mL of either dulanermin or drozitumab for 24 h under both hypoxic (1% O2) and normoxic conditions. Drozitumab was cross‐linked with an anti‐human IgG Fc*γ* for 30 min at 4°C prior to treatment before all in vitro experiments. Crystal Violet staining was used to determine cell viability and optical density was measured at 570 nm wavelength (OD570). Results of representative experiments are presented as the mean ± SD which were performed in triplicate and repeated at least three times.

### Apoptosis analysis

#### Measurement of DEVD‐caspase activity with and without caspase inhibitor 1, ZVAD‐fmk

DEVD‐caspase activity was assayed by cleavage of the fluorogenic substrate zDEVD‐AFC and based on the peptide sequence at the caspase‐3 cleavage site of poly (ADP‐ribose) polymerase. Cells were grown in 96‐well plates at a density of 1 × 10^4^/well and treated for 24 h as indicated, washed once with PBS, and resuspended in 30 *μ*L lysis buffer containing 5 mmol/L EDTA, 5 mmol/L Tris‐HCl, and 10% Igepal (pH 7.5). Cell lysate containing 20 *μ*g of protein was added to each well containing 8 *μ*mol/L substrate in 1 mL fluorometric protease buffer which contained 10% sucrose, 50 mmol/L HEPES, 0.1% CHAPS (pH 7.4), 10 mmol/L DTT. Fluorescence was then quantified (Ex 400 and Em 505) after 4 h at room temperature using a BMG FLUOstar OPTIMA microplate reader. Results were expressed relative to the protein concentration of the sample, which was determined using a commercial BCA protein assay reagent from Thermo Fisher Scientific (Waltham, MA). Caspase Inhibitor 1, ZVAD‐fmk, was resuspended at a concentration of 50 mM and added to the cells at 50 *μ*mol/L alone, with evofosfamide at 50 *μ*mol/L, drozitumab + anti‐human IgG Fc*γ*, or dulanermin at 100 ng/mL.

### Western blot analysis

Cells were treated with 50 *μ*mol/L of evofosfamide, alone or in combination with 100 ng/mL of either dulanermin or drozitumab (cross‐linked with an anti‐human IgG Fc*γ*), under hypoxic (1% O_2_) and normoxic (21% O_2_) conditions for 24 h and lysed in buffer containing 150 mmol/L NaCl, 10 mmol/L Tris‐HCl (pH 7.6), 0.1% sodium dodecyl sulfate, 1% Triton X‐100, 2 mmol/L sodium vanadate, and a protease inhibitor tablet (Roche Diagnostics, Mannheim, Germany). Protein lysates were heated for 10 min at 70°C and loaded under reducing conditions into 4–12% polyacrylamide gels for electrophoresis. Separated proteins were transferred to PVDF membranes (GE Healthcare, Buckinghamshire, UK) electrophoretically and blocked in PBS containing 5% blocking reagent (GE Healthcare, Buckinghamshire, UK) and 0.1% Tween 20 for 1 h at room temperature.

Immunodetection was performed at 4°C overnight in blocking reagent/PBS, using the following primary antibodies mAb anti‐caspase‐8, pAb anti‐caspase‐9, mAb anti‐caspase‐3, and pAb anti‐bid which were purchased from Cell Signaling Technology (Beverly, MA), pAb anti‐Inhibitor of Apoptosis 2 (cIAP2), pAb anti‐Inhibitor of Apoptosis 1 (cIAP1), pAb anti‐XIAP, pAb death receptor 4 (DR4), and pAb death receptor 5 (DR5) purchased from R&D systems, pAb anti‐Poly‐(ADP‐Ribose) Polymerase (PARP) from Roche Diagnostics (Mannheim, Germany). Anti‐actin mAb was used as a loading control and was purchased from SIGMA, Saint Louis, Missouri, USA. All primary antibodies were used at the dilutions suggested by their manufacturers. Membranes were then rinsed three times with PBS containing 0.1% Tween‐20 and incubated for 1 h with a 1:5,000 dilution of anti‐goat, anti‐mouse, or anti‐rabbit alkaline phosphatase‐conjugated secondary antibodies (Thermo Fisher Scientific, Waltham, MA). The ECF substrate reagent kit (GE Healthcare, Buckinghamshire, UK) and the FluorImager (Molecular Dynamics Inc., Sunnyvale, CA) were used to visually assess and quantify the protein bands.

### Animals

For a minimum period of 1 week prior to the commencement of experimentation, 4‐week‐old female athymic mice were acclimatized to the animal housing facility under pathogen‐free conditions (Institute of Medical and Veterinary Services Division, Gilles Plains, SA, Australia). Throughout the experiments, the general physical well‐being and weight of animals were monitored. All experimental procedures on animals were carried out with strict adherence to the guidelines and rules for the ethical use of animals in research and were approved by the Animal Ethics Committees of the Institute of Medical and Veterinary Science and the University of Adelaide, SA, Australia.

### Intratibial injections of osteosarcoma cells

The BTK‐143‐TGL OS cell line was cultured as described previously until 70–80% confluency was reached. Cells were removed from flasks with 2 mmol/L EDTA and resuspended at 1 × 10^5^ cells per 10 *μ*L PBS and kept on ice in an Eppendorf tube. The left tibia was wiped with 70% ethanol and with the knee flexed, coupled to a Hamilton syringe, a 27 gauge needle was inserted through the tibial plateau and 1 × 10^5^ BTK‐143‐TGL cells resuspended in 10 *μ*L of PBS were injected in the marrow space. As the control, all animals were injected with PBS into the contralateral tibia. Mice were randomly assigned into groups of seven animals and 7 days after cancer cell transplantation, drug dosing started. Evofosfamide was administered via i.p injection once a day for 5 days followed by 2 days of rest at 50 mg/kg body weight, whereas drozitumab was administered at 3 mg/kg i.p once a week until the end of the experiment.

### In vivo bioluminescent imaging

The IVIS 100 Imaging system (Xenogen, Alameda, CA) was used weekly to perform noninvasive, whole body imaging to monitor the luciferase‐expressing OS cell line BTK‐143‐TGL in mice using 100 *μ*L of the D‐Luciferin (Xenogen Alameda, CA) solution at final dose of 3 mg/20 g mouse body weight, injected i.p. Mice were then gas‐anesthetized with Isoflurane (Faulding Pharmaceuticals, Salisbury, SA, Australia). Images were acquired from the side angle for 0.5–30 sec (representative images are shown at 1 sec) and the Xenogen Living image (Igor Pro version 2.5) software was used to capture and quantify photon emission from mice in photons/sec per cm^2^.

### Microcomputed tomography ex vivo analysis

The SkyScan‐1072 high‐resolution *μ*CT Scanner (Kontich, Skyscan, Belgium) was operated at 80 kV, 120 *μ*A, rotation step 0.675, with a 0.5 mm Al filter and scan resolution of 5.2 *μ*m/pixel was used to scan surgically resected limbs. Cross sections of the samples were reconstructed using a cone‐beam algorithm (software Cone rec, Skyscan). The growth plate was identified using the 2D images obtained from the *μ*CT scan and starting from the growth plate/tibial interface and moving down the tibia, 450 sections were selected for quantification. To determine 3D bone morphometric parameters (software CTAn, Skyscan), 3D evaluation was performed on all data sets acquired by selecting total bone of the proximal tibia, the cross sections were reconstructed using a cone‐beam algorithm (software Cone_rec, Skyscan). Files were then imported into CTAn software (Skyscan) for 3D analysis and 3D image generation. All images are viewed and edited using ANT visualisation software (Skyscan).

### Data statistics and analysis

Experiments were performed in triplicate and data presented as mean ± SE. SigmaStat for Windows version 3.0 (Systat Software, Inc., Port Richmond, CA) was used for all statistical analysis using the unpaired Student's *T* test. Spearman Rank correlation coefficient was used to assess the association between two variables and comparisons between groups were assessed using a one‐way ANOVA test. In all cases, *P* < 0.05 was considered statistically significant.

## Results

### Evofosfamide cooperates with drozitumab and dulanermin, displaying increased hypoxia‐selective cytotoxicity against OS cells

Human OS cell lines K‐HOS and BTK‐143 were assessed for their sensitivity to the cytotoxic activity of evofosfamide alone and in combination with a maximum dose of 100 ng/mL of drozitumab or dulanermin for 24 h under normoxic (21% O2) and hypoxic (1% O2) conditions. In both OS cell lines as a single agent, evofosfamide had minimal toxicity under normoxic conditions. In contrast, under hypoxic conditions, evofosfamide dose dependently decreased cell viability in both OS cell lines, with 43% viability for the BTK‐143 cells and 65% viability for the K‐HOS cells at 25 *μ*mol/L. Under normoxic conditions, both OS cell lines were resistant to the cytotoxic activity of drozitumab and dulanermin alone at 100 ng/mL. However, under hypoxic conditions, K‐HOS cells were comparably more sensitive to the cytotoxic activity of both drozitumab and dulanermin alone (39 and 47% viability, respectively), whereas BTK‐143 cells were relatively resistant (94 and 77% viability).

**Figure 1:**
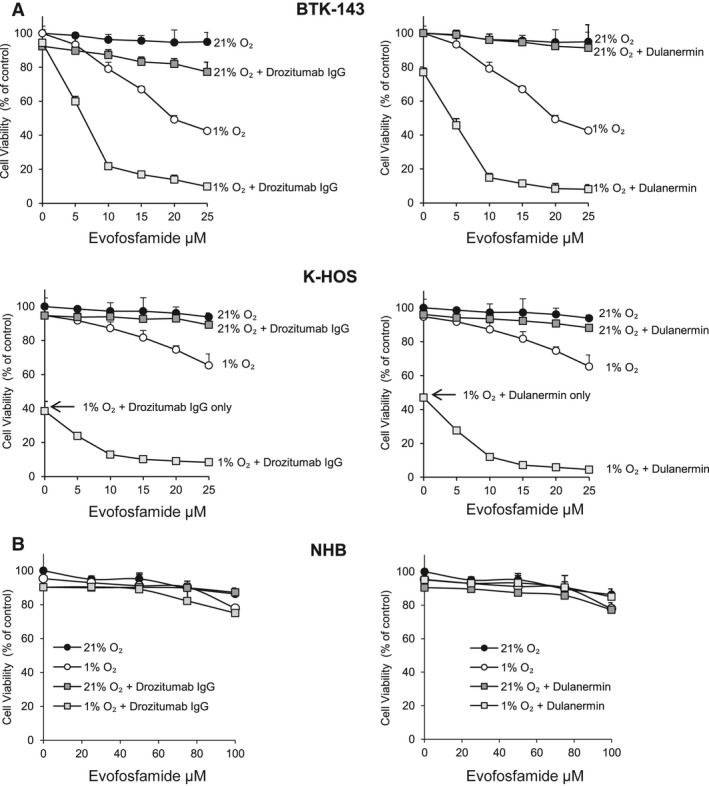
Activity of evofosfamide in combination with drozitumab and dulanermin against OS cells and primary normal human osteoblasts in vitro. (A) OS cell lines BTK‐143 and K‐HOS were seeded in 96‐well plates at 1 × 10^4^ cells per well and treated with increasing doses of evofosfamide alone and in combination with either drozitumab or dulanermin under normoxic (21% O_2_) and hypoxic (1% O_2_) conditions for 24 h. (B) Primary normal human osteoblasts were resistant to evofosfamide and the combination with either drozitumab or dulanermin under the same conditions. Cell viability was assessed by crystal violet staining. Data points show means of quadruplicate results from a representative experiment, repeated at least twice and presented as the mean ± SD of quadruplicate wells and expressed as a percentage of the number of control cells.

Both OS cell lines showed a significant increase in cytotoxicity when either drozitumab or dulanermin were combined with evofosfamide in a dose‐dependent manner under hypoxic conditions, resulting in 95% loss of viability at 25 *μ*mol/L for both OS cell lines (Fig. 1A). In contrast, primary normal human osteoblasts, cultured from patients undergoing hip replacement surgery, were resistant to the cytotoxic activity of evofosfamide at 100 *μ*mol/L in combination with either drozitumab or dulanermin under similar conditions (Fig. 1B).

### Evofosfamide‐mediated OS cytotoxicity is only partly caspase 3‐dependent

**Figure 2:**
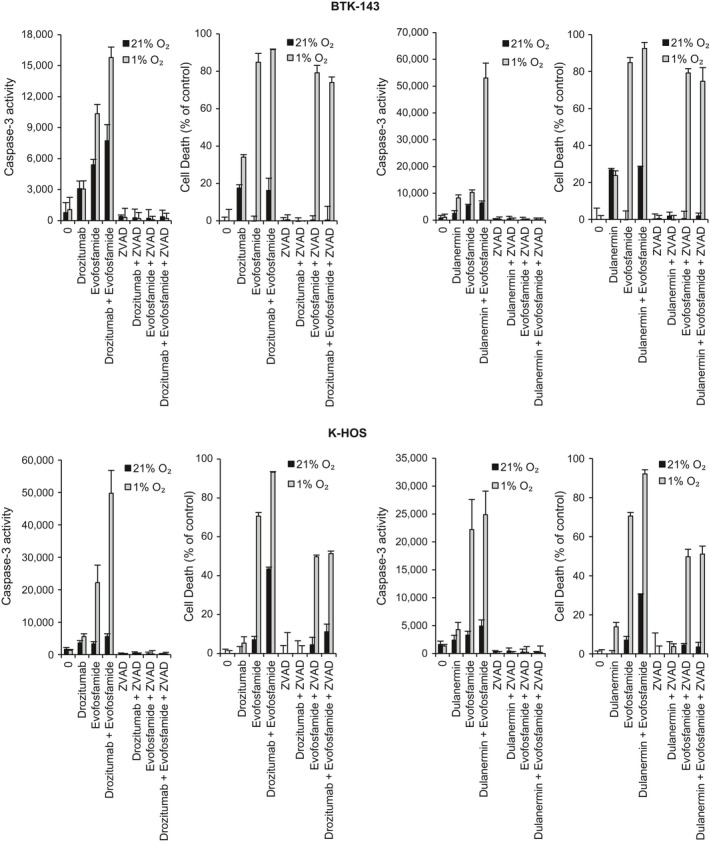
The cytotoxic activity of dulanermin and drozitumab is caspase‐dependent, whereas evofosfamide is not. OS cell lines were seeded in 96‐well plates at 1 × 10^4^ cells per well and treated with evofosfamide alone at 50 *μ*mol/L and with drozitumab IgG 100 ng/mL, dulanermin 100 ng/mL or coincubated with the broad specificity caspase inhibitor z‐VAD‐fmk (50 *μ*mol/L). To exclude possible toxic effects of the inhibitor, cells were also treated with the inhibitor alone under normoxic and hypoxic (1% O_2_) conditions. Cell lysates were used to determine caspase‐3‐like activity, using the caspase‐3‐specific fluorogenic substrate, zDEVD‐AFC and cell viability was assessed via crystal violet staining. Data points show means of quadruplicate results from a representative experiment, repeated at least twice; bars ± SD.

The increase in caspase‐3 activation with 50 *μ*mol/L of evofosfamide treatment under hypoxic conditions alone and in combination with drozitumab or dulanermin (100 ng/mL) was associated with a decrease in cell viability. However, co‐administration with ZVAD‐fmk, a pan‐caspase inhibitor did not prevent the reduction in cell viability caused by evofosfamide in both OS cell lines under hypoxic conditions, despite irreversibly inhibiting the activity of caspase‐3 (Fig. 2), suggesting that the mechanisms involved in evofosfamide‐mediated cytotoxicity are not entirely caspase‐dependent.

However, ZVAD‐fmk completely reversed the cytotoxic activity of both drozitumab and dulanermin in both OS cell lines, indicating that the cytotoxicity of these PARAs against these OS cells is largely caspase dependant, this being in line with the well‐established mechanism of action of these proapoptotic agents. When either drozitumab or dulanermin was combined with evofosfamide and the caspase inhibitor ZVAD‐fmk was added, there was a significant reduction in cytotoxicity against both OS cell lines when compared to the combination of these drugs without ZVAD‐fmk.

**Figure 3:**
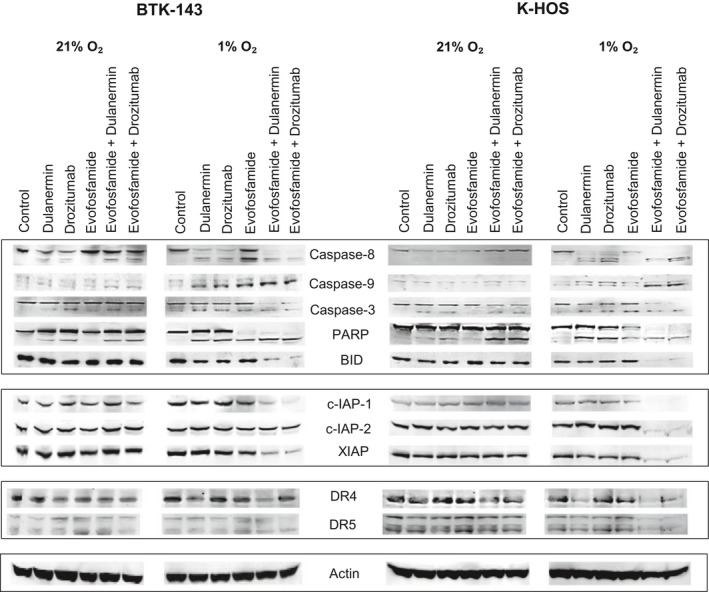
Apoptotic signaling of evofosfamide, dulanermin, and drozitumab against OS cells. OS cells were seeded at 2 × 106 per T25 flask and were treated with evofosfamide at 50 *μ*mol/L, dulanermin and drozitumab at 100 ng/mL under normoxic (21% O2) and hypoxic (1% O2) conditions. After 24 h, cells were lysed and protein was collected. Cell lysates were analyzed by polyacrylamide gel electrophoresis and transferred to PVDF membranes for Immunodetection as described in the Materials and Methods and immunoblotted with various Ab, as shown.

The molecular determinants involved in evofosfamide‐mediated apoptotic signaling alone and in combination with drozitumab or dulanermin were characterized (Fig. 3). Evofosfamide alone treatment at 50 *μ*mol/L under hypoxic conditions (1% O2), for 24 h activated the caspase cascade with robust cleavage of the initiator caspase‐8, caspase‐9, caspase‐3, and cleavage of poly ADP‐ribose polymerase (PARP) which was determined by the detection of the cleaved products in all these antibodies. The mitochondrial proapoptotic Bcl‐2 family protein BID, inhibitor of apoptosis proteins cIAP1, cIAP2, and XIAP, however, remained unchanged. The combination of evofosfamide with dulanermin or drozitumab in both OS cell lines resulted in increased processing of caspases 8, 9, 3, and PARP. Importantly, combination treatment under hypoxia resulted in the robust cleavage of BID, likely resulting in the amplification of apoptotic signaling. Interestingly, we observed a significant decrease in the levels of inhibitor of apoptosis proteins cIAP1, cIAP2, and XIAP in the K‐HOS cell line. The levels of cIAP2 in the BTK‐143 remained unchanged.

Under hypoxic conditions, evofosfamide alone and in combination with either dulanermin or drozitumab upregulated the death receptor DR5 in both cell lines. In the K‐HOS cell line, which was more sensitive to dulanermin and drozitumab when compared to the BTK‐143 cell line, dulanermin and drozitumab alone also upregulated DR5 as single agents under both normoxic and hypoxic conditions. There were no significant differences in the expression of DR4 following treatment.

### Cytotoxic activity of evofosfamide and drozitumab against osteosarcoma‐induced bone destruction

Drozitumab was specifically chosen as opposed to dulanermin in this preclinical study due to its ability to specifically bind to DR5 and not the TRAIL decoy receptors. In addition, drozitumab has a longer half‐life when compared to dulanermin 25.

**Figure 4:**
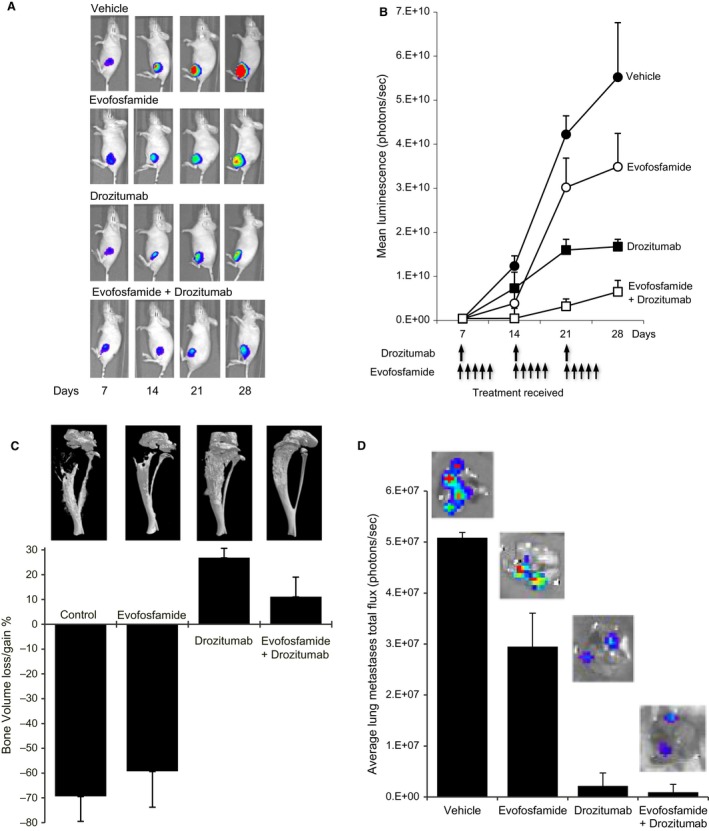
Drozitumab cooperates with evofosfamide to reduce OS intratibial tumors in vivo. BTK‐143‐TGL cells were injected directly into the tibial marrow cavity of 4‐week‐female athymic mice, allowed to establish for 7 days, as described in the methods, mice were imaged weekly using the Xenogen IVIS 100 bioluminescence imaging system. (A). Representative whole body bioluminescent images of a single mouse from each group during the course of the experiment are shown. (B). The line graph, showing average tumor signal over time, expressed as mean photon counts per second during the course of the experiments are shown. Animals receiving treatment with evofosfamide and drozitumab as single agents showed a significant delay in tumor growth. In addition, all mice receiving the combination of evofosfamide and drozitumab showed a further delay of tumor growth when compared with each agent individually. (C). Quantitative assessment of Total bone loss (%) comparing the tumor‐bearing tibiae of each group to the contralateral tibiae and the qualitative 3‐D micro CT images show the osteolytic nature of the BTK‐143‐TGL cell line, which was reduced by drozitumab alone and the combination of evofosfamide and drozitumab. (D). Average lung tumor growth was assessed via bioluminescence showing evofosfamide, drozitumab, and the combination of both agents caused a reduction in lung tumor growth of the BTK‐143‐TGL cell line when compared to the vehicle group. Data shown in each case are the average bioluminescent imaging from all animals in that group: points are means ± SEM.

To investigate the anticancer efficacy of drozitumab and evofosfamide against osteosarcoma progression and metastasis, an orthotopic model of OS was used in which luciferase‐tagged BTK‐143‐TGL cells were directly transplanted into the tibial marrow cavity of female athymic nude mice and accurately monitored and quantified using noninvasive bioluminescence imaging over a 28–day‐period 24. Treatment with drozitumab, evofosfamide, or the combination of both agents commenced 7 days after the intratibial OS cell injections. All vehicle‐treated animals showed an increase in mean photon emission exponentially, which indicated an increase in tumor burden palpable from day 7 onward, reaching a maximum signal at day 28, at which point animals were humanely killed. In contrast, treatment with evofosfamide or drozitumab showed a reduction in tumor burden over the same period in all animals. Importantly, the combination demonstrated a far greater anticancer efficacy in the bone (Fig 4A and B). The tibiae of all mice were dissected at the end of the experiment and the qualitative and quantitative assessment of bone destruction was analyzed using high resolution *μ*CT (Fig 4C). In the vehicle‐treated animals, extensive osteolysis was clearly evident such that the net loss in bone volume (BV) was 69% in the left tumor‐bearing tibiae when compared to the contralateral nontumor bearing right tibiae.

Although tumor burden was reduced by evofosfamide treatment, this did not prevent bone destruction such that the extent of osteolysis was not significantly different when compared to the vehicle‐treated group. Remarkably, treatment with drozitumab alone resulted in extensive bone remodeling, resulting in a gain of bone volume of 27% when compared to the untreated right tibia. Micro‐CT analysis showed extensive bone remodeling that was noticeable under the growth plate and extended down the length of the tibia where the tumor resided. Tumor healing in the treatment of osteosarcoma in patients if often correlated with a significant increase in calcification, which would account for the increase in calcification in the tumor affected tibia of mice treated with drozitumab 26. In animals treated with the combination of drozitumab and evofosfamide, the tibia had a normal appearance due to the full mineralization of the cortical bone, demonstrating additional protection of the bone architecture, less calcification, and more advanced bone remodeling, such that the net gain of BV was reduced to 6%.

Ex vivo bioluminescence imaging showed no differences between the treated groups (three out of seven mice) in the number of mice that developed lung metastases which may be due to cancer cells entering into the blood stream and metastasizing to the lung during the 7 days before treatment. However, the tumor burden in the lungs of the mice with metastases which was measured as a function of bioluminescence signal showed a reduction in tumor growth with evofosfamide treatment. In addition, drozitumab maintained its cytotoxicity against metastatic OS cells in the lungs, which led to the tumor burden being reduced in both the drozitumab and combination groups (Fig 4D).

#### Effect of evofosfamide on bone metabolism

Our experimental approach also provides an opportunity to assess the normal bone parameters after treating the mice with evofosfamide, drozitumab, and the combination of both. After 3 weeks of treatment, the use of high‐resolution micro‐CT analysis to compare the contralateral nontumor‐bearing tibiae of treated and untreated animals showed no differences in any of the microarchitectural bone morphometric parameters, which included total bone volume, bone surface, trabecular number, trabecular thickness, or trabecular spacing (Table 1).

**Table 1:** Comparison of bone morphometric parameters of contralateral nontumor‐injected tibiae from vehicle, evofosfamide‐, drozitumab‐, evofosfamide + drozitumab‐treated animals

Parameters	Vehicle control		Evofosfamide		Drozitumab		Evofosfamide + Drozitumab	
	Mean	SE	Mean	SE	Mean	SE	Mean	SE
Bone volume(mm^3^)	2.34	0.05	2.27	0.07	2.31	0.14	2.30	0.11
Bone surface (mm^2^)	191.68	4.07	194.55	4.94	188.71	6.58	195.52	10.91
Intersection surface (mm^2^)	0.36	0.03	0.32	0.04	0.38	0.03	0.36	0.09
Trabecular space (mm)	1.53	0.02	1.49	0.04	1.57	0.04	1.45	0.03
Trabecular number (1/mm)	0.16	0.07	0.18	0.01	0.16	0.02	0.20	0.02
Trabecular thickness (mm)	0.05	0	0.05	0	0.06	0	0.05	0
Trabecular pattern factor (1/mm)	24.24	1.37	21.17	1.79	23.90	0.77	22.05	1.05
Structure model index	2.14	0.04	2.04	0.23	2.08	0.03	1.92	0.06

Bone volume, bone surface, intersection surface, trabecular space, trabecular number, trabecular thickness, trabecular pattern factor, and structure model index were measured by three‐dimensional analysis of *μ*CT images of the contralateral tibial bone.

Results are expressed as mean ± SE. Significance of results is with respect to untreated animals obtained using Student's *t* test.

## Discussion

In addition to surgical intervention, chemotherapeutic agents such as doxorubicin, etoposide, cisplatin, and cyclophosphamide used alone, or in combination have significantly improved overall survival for patients with OS. Yet, despite these improvements in treating the primary tumor, a large number of patients with OS eventually develop lung metastases, even after surgical excision and conventional chemotherapy. There is a need to therefore, develop safe and new approaches for OS treatment 27, 28, 29.

It must be noted that when compared to other tissues, the bone marrow and in particular the hematopoietic niche close to the endosteal surface is hypoxic, which is required for normal hematopoiesis to occur 30. Unlike soft tissue tumors, OS can also adapt to this hypoxic bone microenvironment. The ability to target OS in this hypoxic bone environment is therefore an important feature that evofosfamide has over other cancer therapies. In addition, conventional chemotherapeutics are usually cytotoxic to normal bone cells in the bone marrow, an important goal of anticancer treatment is to selectively target cancer cells but not normal bone cells.

A combinatorial approach using agents with additive or synergistic cytotoxic activities are appealing because they allow lower drug doses to be used, which reduce harmful side effects, particularly in the bone. Consistent with our previous published data 31, 32 under normoxic conditions, evofosfamide alone resulted in minimal toxicity against OS, whereas under hypoxic conditions, evofosfamide decreased OS cell viability. In addition, under normoxic conditions, both OS cell lines were resistant to the cytotoxic activity of drozitumab and dulanermin as single agents. However, under hypoxic conditions, K‐HOS cells were comparably more sensitive to the cytotoxic activity of both drozitumab and dulanermin alone, while BTK‐143 cells were relatively resistant. This resensitization of the K‐HOS cell line to both these drugs may be attributed to the hypoxic conditions providing an additional stress mechanism, which in turn activate the extrinsic and intrinsic apoptotic pathways for this OS cell line. Importantly, while both OS cell lines are resistant to the treatments under normoxic conditions, under hypoxic conditions, this cytotoxic activity was further increased when evofosfamide was co administered with either drozitumab or dulanermin under hypoxic conditions. The combination of the chemotherapeutic agents’ drozitumab and dulanermin with evofosfamide was not toxic to either normal human bone cells in vitro or normal bone metabolism in vivo, corroborating with previous studies which demonstrate that these agents individually are nontoxic to normal bone. 24, 31, 33. These results highlight not only the hypoxic selectivity of evofosfamide, but also the specific tumor selectivity of both evofosfamide and PARAs.

In the search for more effective treatments for OS, PARAs including recombinant dulanermin and the agonistic antibody drozitumab induce apoptosis through different but overlapping signaling pathways, whereas evofosfamide induces apoptosis mainly through caspase‐independent mechanisms as described previously 34. As a result, the combination of PARAs and evofosfamide were considerably more cytotoxic to tumor cells that resist cytotoxic activity through a single pathway, where inhibiting caspase activity to prevent the activity of both drozitumab and dulanermin still resulted in both OS cell lines under hypoxic conditions being sensitive to the cytotoxic activity of evofosfamide. This is also reflected by the activation of caspase‐8, caspase‐9, caspase‐3, PARP, cleavage of Bid, a member of the Bcl‐2 family protein, and the downregulation of c‐IAP1 when evofosfamide was combined with dulanermin or drozitumab as well as both PARAs activating the extrinsic pathway by the upregulation of DR5.

Based on our in vitro results, the therapeutic potential of evofosfamide was expected to be greatest in combination with adjuvant cytotoxic chemotherapy. When transplanted into the tibial marrow cavity of mice, BTK‐143 cells are highly osteolytic and this results in extensive bone destruction and the development of metastases to the lung 3–4 weeks post cancer cell transplantation. This in vivo model mimics OS activity in the bone as seen in patients with the disease and is ideal for determining the potential of drug treatment on cancer growth in the bone as well as cancer‐induced bone destruction 24, 31. The activity of evofosfamide in combination with drozitumab was tested in this context, in a preclinical model of OS progression and development for the following reasons. In contrast to dulanermin, which has a short bioavailability of 30 min, which requires daily treatment for patients and the inability to bind to death‐inducing TRAIL receptors in various cancer types, preferring to bind with the decoy TRAIL receptors 35, 36, drozitumab is a fully agonistic human monoclonal antibody that specifically binds to and activates DR5 in the same manner as dulanermin 24. Drozitumab has a half‐life ranging from several days to weeks and has been developed to specifically target DR5 37 and not the TRAIL decoy receptors. In addition, circulating Fragment Crystalline Gamma (Fc*γ*) receptors expressed on the surface of various immune cells 38, 39, crosslink with drozitumab which leads to enhanced antibody‐dependent, cell‐mediated cytotoxicity (ADCC) 40, resulting in immune cell activation leading to recruitment of other Fc*γ* receptor‐expressing cells to the tumor microenvironment 36, 40. The apoptotic tumor cells are then phagocytosed by the activated Fc*γ* receptor‐expressing immune cells 41, further enhancing the cytotoxic activity of drozitumab against cancer.

The activity of drozitumab against OS in bone has yet to be reported and in addition, this OS cell line is relatively resistant to drozitumab in vitro, allowing the detection of any synergistic or additive activity to be easily observed.

As a single agent, evofosfamide had limited impact in reducing tumor growth in the tibia or protecting the tibia from the cancer‐induced bone destruction caused by this highly aggressive osteolytic cell line.

The cytotoxic activity displayed by drozitumab in vivo contradicts the resistance of this human osteosarcoma shown in vitro. A possible explanation to account for the increase in cytotoxicity of drozitumab in vivo is the circulation of Fc*γ* receptors expressed by leukocytes in mice. The engagement of leukocyte Fc*γ* receptors by antibody‐antigen complexes leads to an enhanced antibody‐dependent, cell‐mediated cytotoxicity (ADCC) 40, which can interact more efficiently with the DR5 agonistic antibody drozitumab when compared to artificial Fc crosslinking in vitro, leading to improved cytotoxicity against the human osteosarcoma in the tibia and lungs of the mice.

The combination of both evofosfamide and drozitumab had a profound effect in preventing growth of the tumor within the tibia which also translated to increased bone protection and a reduction in tumor burden in the lung. This may be related to the ability of evofosfamide to upregulate DR5 expression under hypoxic conditions, resulting in increased sensitivity to Drozitumab as observed in previous studies 42. In addition, each drug specifically targets tumor regions of different oxygen tensions accordingly.

PARAs including drozitumab and dulanermin have been tested either alone or in combination with other agents in phase I and II clinical trials 19, with little clinical benefit observed to date which has led to the discontinuation of the development of PARAs in many cases 43. However, none of these clinical trials have examined the anticancer efficacy of PARAs against cancers in the bone such as OS.

Evofosfamide is currently being evaluated both as monotherapy and in combination with conventional chemotherapy and radiotherapy in numerous phase I and phase II clinical trials against a variety of cancer types. To date, two phase 3 trials targeting unresectable or metastatic soft tissue sarcoma NCT01440088 and unresectable pancreatic adenocarcinoma NCT01746979 43 failed to meet their primary endpoint of improving overall survival with statistical significance. Nonetheless, from the observations in phase I and II clinical trials of evofosfamide and PARAs, and from the results presented in this study, which indicate that these compounds are nontoxic to normal bone metabolism suggest that OS patients may benefit from evofosfamide when used in combination with PARAs.

## Conflict of Interest

None declared.

## References

[1] Campanacci, M. 1999 Bone and Soft Tissue Tumors, 2nd ed. Piccin Nuova Libraria, Padova.

[2] Pringle, J. A. S. 1999 Bone‐forming neoplasms arising within bone in Helliwell T. R., ed. Pathology of bone and joint neoplasms. Saunders, Philadelphia.

[3] Tang, N. , W. X. Song , J. Y. Luo , R. C. Haydon , and T. C. He . 2008 Osteosarcoma development and stem cell differentiation. Clin. Orthop. Relat. Res. 466:2114–2130. 18563507 10.1007/s11999-008-0335-z PMC2492997

[4] Link, M. P. , A. M. Goorin , M. Horowitz , W. H. Meyer , J. Belasco , A. Baker , et al. 1991 Adjuvant chemotherapy of high‐grade osteosarcoma of the extremity. Updated results of the Multi‐Institutional Osteosarcoma Study. Clin. Orthop. Relat. Res. 8–14. 1884563

[5] Saeter, G. , T. A. Alvegard , I. Elomaa , T. Wiebe , O. Bjork , H. Strander , et al. 1997 Chemotherapy for osteosarcoma and Ewing's sarcoma. Acta Orthop. Scand. 68:120–125. 10.1080/17453674.1997.11744716 9057601

[6] Chan, H. S. , T. M. Grogan , G. Haddad , G. DeBoer , and V. Ling . 1997 P‐glycoprotein expression: critical determinant in the response to osteosarcoma chemotherapy. J. Natl Cancer Inst. 89:1706–1715. 9390540 10.1093/jnci/89.22.1706

[7] Gralow, J. R. , J. S. Biermann , A. Farooki , M. N. Fornier , R. F. Gagel , R. N. Kumar , et al. 2009 NCCN task force report: bone health in cancer care. J. Natl. Compr. Canc. Netw. 7:S1–S32. 10.6004/jnccn.2009.0076 PMC3047404 19555589

[8] Lustberg, M. B. , R. E. Reinbolt , and C. L. Shapiro . 2012 Bone health in adult cancer survivorship. J. Clin. Oncol. 30:3665–3674. 23008309 10.1200/JCO.2012.42.2097

[9] Mundy, G. R. 2002 Metastasis to bone: causes, consequences and therapeutic opportunities. Nat. Rev. Cancer 2:584–593. 12154351 10.1038/nrc867

[10] Goltzman, D. 2001 Osteolysis and cancer. J. Clin. Invest. 107:1219–1220. 11375409 10.1172/JCI13073 PMC209307

[11] Taube, T. , I. Elomaa , C. Blomqvist , M. N. Beneton , and J. A. Kanis . 1994 Histomorphometric evidence for osteoclast‐mediated bone resorption in metastatic breast cancer. Bone 15:161–166. 8086233 10.1016/8756-3282(94)90703-x

[12] Chirgwin, J. M. , and T. A. Guise . 2000 Molecular mechanisms of tumor‐bone interactions in osteolytic metastases. Crit. Rev. Eukaryot. Gene Expr. 10:159–178. 11186331

[13] Goltzman, D. 1997 Mechanisms of the development of osteoblastic metastases. Cancer 80(8 Suppl):1581–1587. 9362425 10.1002/(sici)1097-0142(19971015)80:8+<1581::aid-cncr8>3.3.co;2-8

[14] Goltzman, D. , A. C. Karaplis , R. Kremer , and S. A. Rabbani . 2000 Molecular basis of the spectrum of skeletal complications of neoplasia. Cancer 88(12 Suppl):2903–2908. 10898332 10.1002/1097-0142(20000615)88:12+<2903::aid-cncr4>3.0.co;2-g

[15] Duan, J.‐X. , H. Jiao , J. Kaizerman , T. Stanton , James. W. Evans , L. Lan , et al. 2007 Potent and highly selective hypoxia‐activated achiral phosphoramidate mustards asanticancer drugs. J. Med. Chem. 51:2412–2420. 10.1021/jm701028q 18257544

[16] Chawla, S. P. , L. D. Cranmer , B. A. Van Tine , D. R. Reed , S. H. Okuno , J. E. Butrynski , et al. 2014 Phase II study of the safety and antitumor activity of the hypoxia‐activated prodrug TH‐302 in combination with doxorubicin in patients with advanced soft tissue sarcoma. J. Clin. Oncol. 32:3299–3306. 25185097 10.1200/JCO.2013.54.3660 PMC4588714

[17] Borad, M. J. , S. G. Reddy , N. Bahary , H. E. Uronis , D. Sigal , A. L. Cohn , et al. 2014 Randomized phase II trial of gemcitabine plus TH‐302 versus gemcitabine in patients with advanced pancreatic cancer. J. Clin. Oncol. 33:1475–1481. 25512461 10.1200/JCO.2014.55.7504 PMC4881365

[18] Wojtkowiak, J. W. , H. C. Cornnell , S. Matsumoto , K. Saito , Y. Takakusagi , P. Dutta , et al. 2015 Pyruvate sensitizes pancreatic tumors to hypoxia‐activated prodrug TH‐302. Cancer Metabol. 3:2. 10.1186/s40170-014-0026-z PMC4310189 25635223

[19] Dine, J. L. , C. C. O'Sullivan , D. Voeller , Y. E. Greer , K. J. Chavez , C. M. Conway , et al. 2016 The TRAIL receptor agonist drozitumab targets basal B triple‐negative breast cancer cells that express vimentin and Axl. Breast Cancer Res. Treat. 155:235–251. 26759246 10.1007/s10549-015-3673-z PMC4753803

[20] Soria, J.‐C. , E. Smit , D. Khayat , B. Besse , X. Yang , C.‐P. Hsu , et al. 2010 Phase 1b study of dulanermin (recombinant human Apo2L/TRAIL) in combination with paclitaxel, carboplatin, and bevacizumab in patients with advanced non‐squamous non‐small‐cell lung cancer. J. Clin. Oncol. 28:1527–1533. 20159815 10.1200/JCO.2009.25.4847

[21] Younes, A. , J. M. Vose , A. D. Zelenetz , M. R. Smith , H. A. Burris , S. M. Ansell , et al. 2010 A Phase 1b/2 trial of mapatumumab in patients with relapsed/refractory non‐Hodgkin's lymphoma. Br. J. Cancer 103:1783–1787. 21081929 10.1038/sj.bjc.6605987 PMC3008610

[22] Kindler, H. L. , D. A. Richards , L. E. Garbo , E. B. Garon , J. J. Jr Stephenson , C. M. Rocha‐Lima , et al. 2012 A randomized, placebo‐controlled phase 2 study of ganitumab (AMG 479) or conatumumab (AMG 655) in combination with gemcitabine in patients with metastatic pancreatic cancer. Ann. Oncol. 23:2834–2842. 22700995 10.1093/annonc/mds142

[23] Herbst, R. S. , S. G. Eckhardt , R. Kurzrock , S. Ebbinghaus , P. J. O'Dwyer , M. S. Gordon , et al. 2010 Phase I dose‐escalation study of recombinant human Apo2L/TRAIL, a dual proapoptotic receptor agonist, in patients with advanced cancer. J. Clin. Oncol. 28:2839–2846. 20458040 10.1200/JCO.2009.25.1991

[24] Zinonos, I. , A. Labrinidis , M. Lee , V. Liapis , S. Hay , V. Ponomarev , et al. 2009 Apomab, a fully human agonistic antibody to DR5, exhibits potent antitumor activity against primary and metastatic breast cancer. Mol. Cancer Ther. 8:2969–2980. 19808976 10.1158/1535-7163.MCT-09-0745 PMC5568046

[25] Ashkenazi, A. , P. Holland , and S. G. Eckhardt . 2008 Ligand‐based targeting of apoptosis in cancer: the potential of recombinant human apoptosis ligand 2/tumor necrosis factor‐related apoptosis‐inducing ligand (rhApo2L/TRAIL). J. Clin. Oncol. 26:3621–3630. 18640940 10.1200/JCO.2007.15.7198

[26] Ferrari, S. , A. Balladelli , E. Palmerini , and D. Vanel . 2013 Imaging in bone sarcomas. The chemotherapist's point of view. Eur. J. Radiol. 82:2076–2082. 22209429 10.1016/j.ejrad.2011.11.028

[27] He, J.‐P. , Y. Hao , Y. Hao , Wang, X.‐L. , Yang, X.‐J. , Shao, J.‐F. , et al. 2014 Review of the molecular pathogenesis of osteosarcoma. Asian Pac. J. Cancer Prev. 15:5967–5976. 25124559 10.7314/apjcp.2014.15.15.5967

[28] Yang, J. , and W. Zhang . 2013 New molecular insights into osteosarcoma targeted therapy. Curr. Opin. Oncol. 25:398–406. 23666471 10.1097/CCO.0b013e3283622c1b

[29] Botter, S. M. , D. Neri , and B. Fuchs . 2014 Recent advances in osteosarcoma. Curr. Opin. Pharmacol. 16:15–23. 24632219 10.1016/j.coph.2014.02.002

[30] Miharada, K. , G. Karlsson , M. Rehn , E. Rorby , K. Siva , J. Cammenga , et al. 2011 Cripto regulates hematopoietic stem cells as a hypoxic‐niche‐related factor through cell surface receptor GRP78. Cell Stem Cell 9:330–344. 21982233 10.1016/j.stem.2011.07.016

[31] Liapis, V. , A. Labrinidis , I. Zinonos , S. Hay , V. Ponomarev , V. Panagopoulos , et al. 2015 Hypoxia‐activated pro‐drug TH‐302 exhibits potent tumor suppressive activity and cooperates with chemotherapy against osteosarcoma. Cancer Lett. 357:160–169. 25444931 10.1016/j.canlet.2014.11.020 PMC4574867

[32] Liapis, V. , I. Zinonos , A. Labrinidis , S. Hay , V. Ponomarev , V. Panagopoulos , et al. 2016 Anticancer efficacy of the hypoxia‐activated prodrug evofosfamide (TH‐302) in osteolytic breast cancer murine models. Cancer Med. 5:534–545. 26749324 10.1002/cam4.599 PMC4799961

[33] Labrinidis, A. , P. Diamond , S. Martin , S. Hay , V. Liapis , I. Zinonos , et al. 2009 Apo2L/TRAIL inhibits tumor growth and bone destruction in a murine model of multiple myeloma. Clin. Cancer Res. 15:1998–2009. 19276263 10.1158/1078-0432.CCR-08-2444 PMC5573683

[34] Ashkenazi, A. , R. C. Pai , S. Fong , S. Leung , D. A. Lawrence , S. A. Masters , et al. 1999 Safety and antitumor activity of recombinant soluble Apo2 ligand. J. Clin. Invest. 104:155–162. 10411544 10.1172/JCI6926 PMC408479

[35] Amarante‐Mendes, G. P. , and T. S. Griffith . 2015 Therapeutic applications of TRAIL receptor agonists in cancer and beyond. Pharmacol. Ther. 155:117–131. 26343199 10.1016/j.pharmthera.2015.09.001 PMC4955572

[36] Ashkenazi, A. 2008 Directing cancer cells to self‐destruct with pro‐apoptotic receptor agonists. Nat. Rev. Drug Discovery 7:1001–1012. 18989337 10.1038/nrd2637

[37] Holland, P. M. 2013 Targeting Apo2L/TRAIL receptors by soluble Apo2L/TRAIL. Cancer Lett. 332:156–162. 21220186 10.1016/j.canlet.2010.11.001

[38] Robak, T. 2013 Emerging monoclonal antibodies and related agents for the treatment of chronic lymphocytic leukemia. Future Oncol. 9:69–91. 23252565 10.2217/fon.12.157

[39] Wilson, N. S. , B. Yang , A. Yang , S. Loeser , S. Marsters , D. Lawrence , et al. 2011 An Fc gamma receptor‐dependent mechanism drives antibody‐mediated target‐receptor signaling in cancer cells. Cancer Cell 19:101–113. 21251615 10.1016/j.ccr.2010.11.012

[40] Takeda, K. , N. Yamaguchi , H. Akiba , Y. Kojima , Y. Hayakawa , J. E. Tanner , et al. 2004 Induction of tumor‐specific T cell immunity by anti‐DR5 antibody therapy. J. Exp. Med. 199:437–448. 14769851 10.1084/jem.20031457 PMC2211825

[41] Alexiou, G. A. , K. I. Tsamis , and A. P. Kyritsis . 2015 Targeting tumor necrosis factor‐related apoptosis‐inducing ligand (TRAIL): a promising therapeutic strategy in Gliomas. Semin. Peadiatr. Neurol. 22:35–39. 10.1016/j.spen.2014.12.002 25976259

[42] Holland, P. M. 2014 Death receptor agonist therapies for cancer, which is the right TRAIL? Cytokine Growth Factor Rev. 25:185–193. 24418173 10.1016/j.cytogfr.2013.12.009

[43] Alama, A. , A. M. Orengo , S. Ferrini , and R. Gangemi . 2012 Targeting cancer‐initiating cell drug‐resistance: a roadmap to a new‐generation of cancer therapies? Drug Discovery Today 17(9–10):435–442. 21315830 10.1016/j.drudis.2011.02.005

